# Supplementary material for: Nanostructured Iron Sulfide/N, S Dual-Doped Carbon Nanotube-Graphene Composites as Efficient Electrocatalysts for Oxygen Reduction Reaction
Source: Materials (Basel). 2021 Apr 23;14(9):2146. doi: 10.3390/ma14092146 (PMC8122905; doi:10.3390/ma14092146)
Supplement: Supplementary file 1 [file materials-14-02146-s001.zip › materials-1198849-supplementary.pdf]

materials

MDPI

Supplementary Materials

Nanostructured Iron Sulfide/N, S Dual-Doped Carbon Nanotube-Graphene Composites as Efficient Electrocatalysts for Oxygen Reduction Reaction

Gyu Sik Chae [1], Duck Hyun Youn [2,*] and Jae Sung Lee [3,*]

[1] Division of Environmental Science and Engineering, Pohang University of Science and Technology (POSTECH), Pohang 37673, Korea; she213@postech.ac.kr
[2] Department of Chemical Engineering, Interdisciplinary Program in Advanced Functional Materials and Devices Development, Kangwon National University, Chuncheon, Gangwon-do 24341, Korea
[3] School of Energy & Chemical Engineering, Ulsan National University of Science and Technology (UNIST), Ulsan 44919, Korea
* Correspondence: youndh@kangwon.ac.kr (D.H.Y.); jlee1234@unist.ac.kr (J.S.L.)

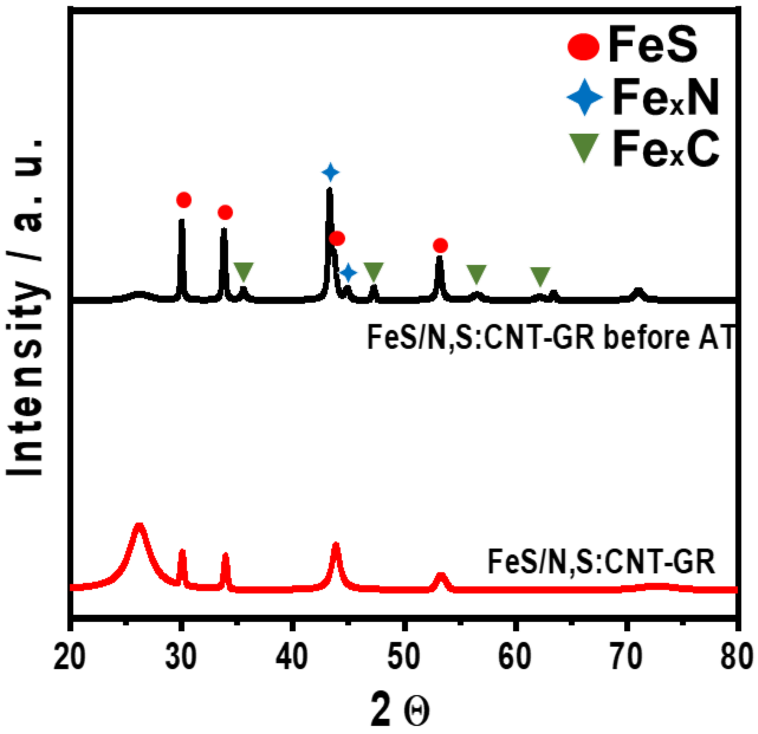

Figure S1. XRD patterns of FeS/N,S:CNT-GR catalyst before and after acid treatment.

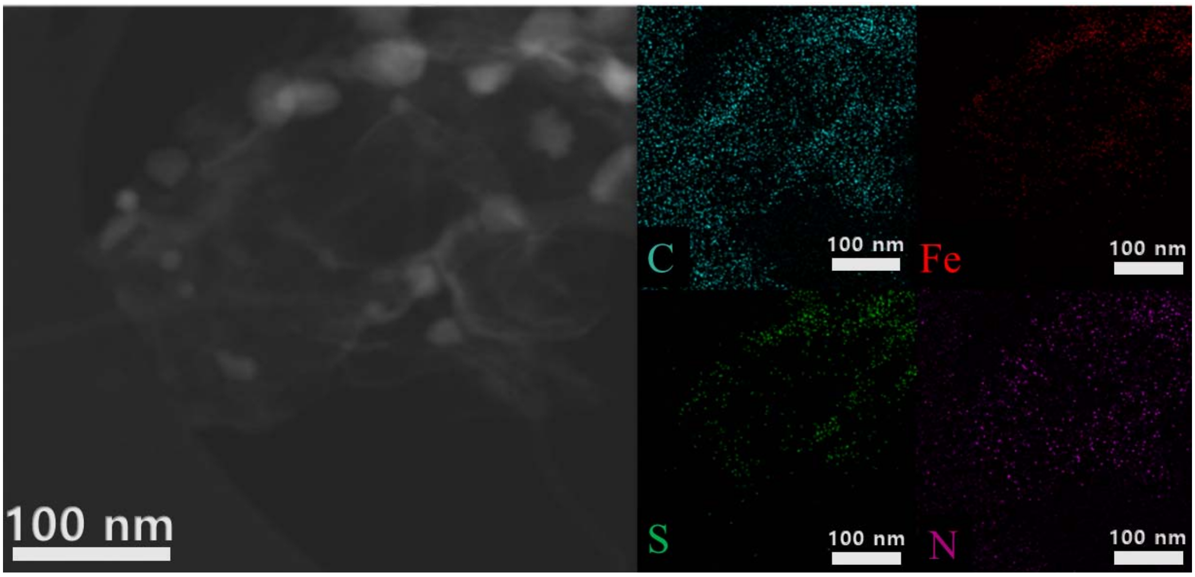

Figure S2. EDS elemental mapping images of FeS/N,S:CNT-GR for C, Fe, S, and N.

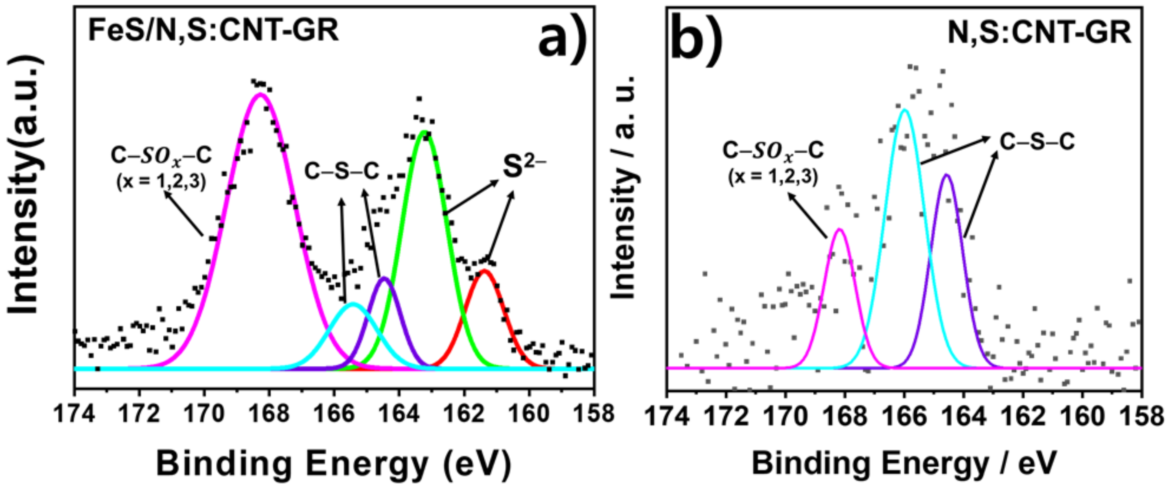

Figure S3. XPS S 2p spectra of (**a**) FeS/N,S:CNT-GR and (**b**) N,S:CNT-GR.

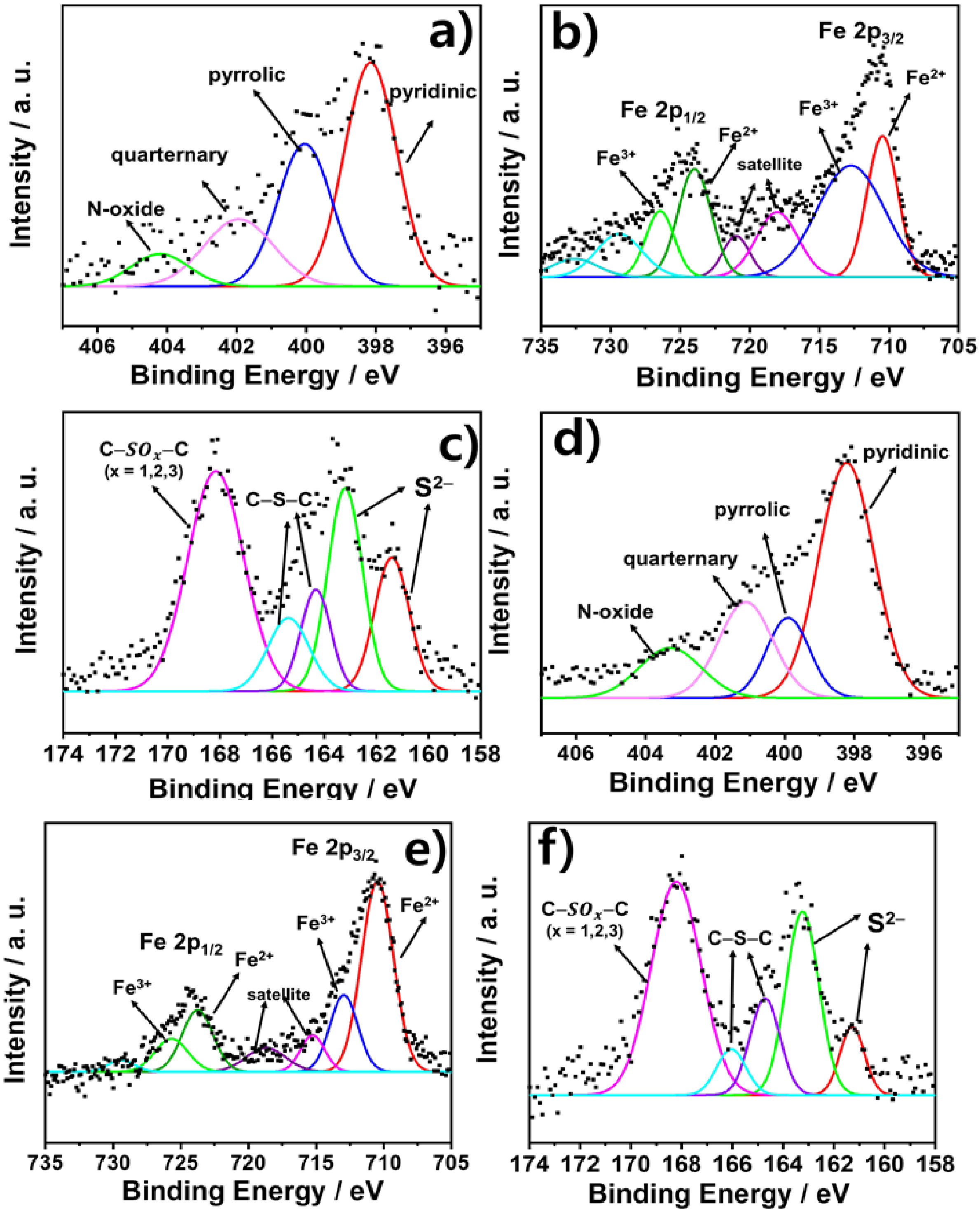

Figure S4. XPS spectra of FeS/N,S:CNT for (**a**) N 1s, (**b**) Fe 2p, and (**c**) S 2p. XPS spectra of FeS/N,S:GR for (**d**) N 1s, (**e**) Fe 2p, and (**f**) S 2p.

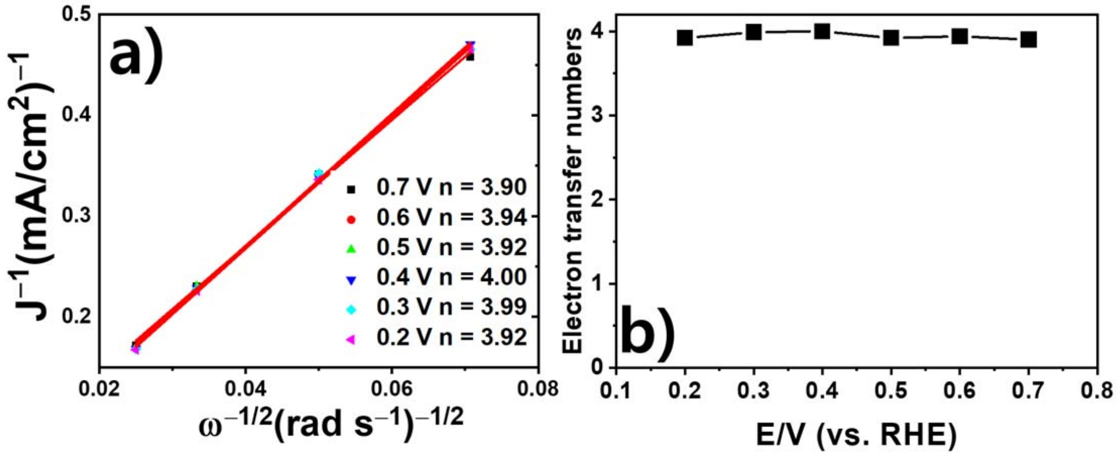

Figure S5. (**a**) Koutecky-Levich plots and (**b**) corresponding electron transfer number of FeS/N,S:CNT-GR at different potentials.

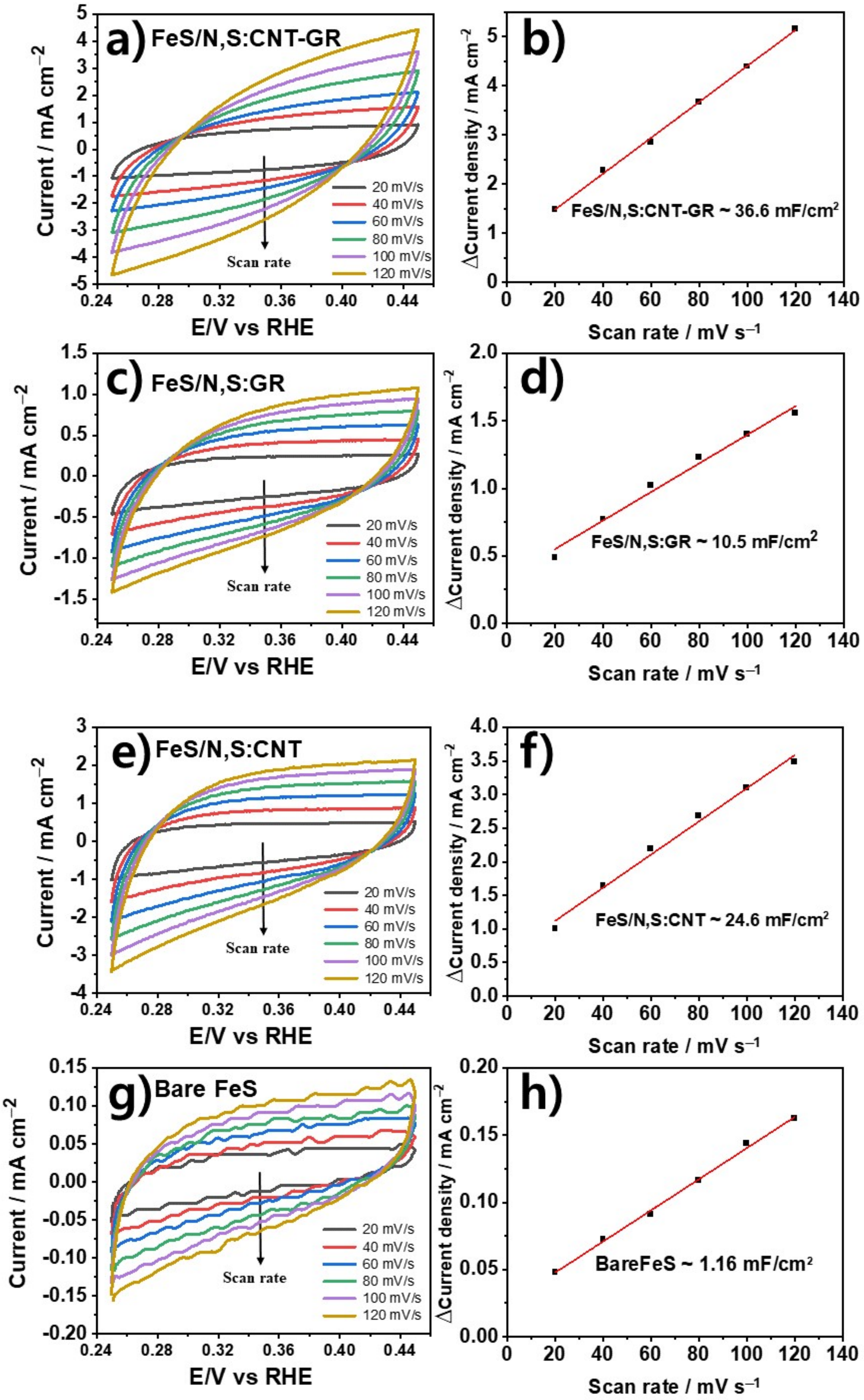

Figure S6. Cyclic voltammograms at different scan rates in N_2-saturated KOH solution and C_{dl} calculation of (**a**,**b**) FeS/N,S:CNT-GR, (**c**,**d**) FeS/N,S:GR, (**e**,**f**) FeS/N,S:CNT, and (**g**,**h**) bare FeS.

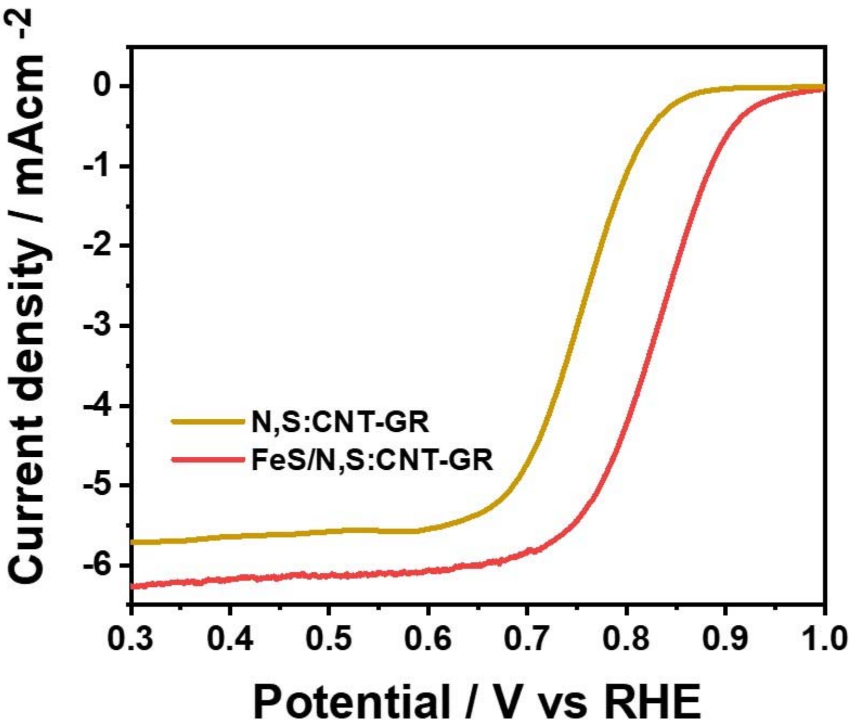

Figure S7. LSV curves of FeS/N,S:CNT-GR and N,S:CNT-GR.

Table S1. Comparison of BET surface area, pore volume, and average pores size of the prepared catalysts with TMS-based electrocatalysts.

Catalysts	BET Surface Areas (m2·g^{-1})	Pore Volume (cm^3·g^{-1})	Pore Size (nm)	References
FeS/N,S:CNT-GR	191	0.5568	4	This work
FeS/N,S:CNT	174	0.5067	4	This work
FeS/N,S:GR	137	0.4077	4	This work
FeS/G (Fe:GO = 1:4)	334.15	0.91	4	1
FeS/G (Fe:GO = 1:1)	76.45	0.845	3.5	1
FeS_2-RGO	70.59	-	4	2
ECA-CNS (cobalt nikel sulfide)	71.4	-	-	3
Co_9S_8/N,S-CNS (carbon nanosheet)	22	-	10	4
CoS_2(400)/N,S-GO	19	-	3.5	5

Supplementary References

1. Gautam, J.; Tran, D.T.; Singh, T.I.; Kim, N.H.; Lee, J.H. Mesoporous iron sulfide nanoparticles anchored graphene sheet as an efficient and durable catalyst for oxygen reduction reaction. *J. Power Sources* **2019**, *427*, 91–100.
2. Jiang, J.; Zhu, L.; Chen, H.; Sun, Y.; Qian, W.; Lin, H.; Han, S. Highly active and stable electrocatalysts of FeS 2–reduced graphene oxide for hydrogen evolution. *J. Mater. Sci.* **2019**, *54(2)* ,1422–1433.
3. Hong, Y. R.; Mhin, S.; Kim, K. M.; Han, W. S.; Choi, H.; Ali, G.; Chung, K.Y.; Lee, H.J.; Moon, S.I.; Dutta, S.; Sun, S.H.; Jung, Y.G.; Song, T.S.; Han, H. Electrochemically activated cobalt nickel sulfide for an efficient oxygen evolution reaction: partial amorphization and phase control. *J. Mater. Chem. A* **2019**, *7(8),* 3592–3602.
4. Wu, C.; Zhang, Y.; Dong, D.; Xie, H.; Li, J. Co 9 S 8 nanoparticles anchored on nitrogen and sulfur dual-doped carbon nanosheets as highly efficient bifunctional electrocatalyst for oxygen evolution and reduction reactions. *Nanoscale* **2017**, *9*, 12432–12440.
5. Ganesan, P.; Prabu, M.; Sanetuntikul, J.; Shanmugam, S. Cobalt sulfide nanoparticles grown on nitrogen and sulfur co-doped graphene oxide: An efficient electrocatalyst for oxygen reduction and evolution reactions, *ACS Catal* **2015**, *5,* 3625–3637.

Table S2. Comparison of ORR performance in 0.1 M KOH electrolyte of FeS/N,S:CNT-GR with other iron or TMS-based electrocatalysts.

Catalysts	Electrolyte	Onset Potential (vs. RHE)	Half Potential (vs. RHE)	Current Density at 0.3V (mA cm^{-2})	References
FeS/N,S:CNT-GR	0.1 M KOH	0.972	0.827	−6.2	This work
FP-Fe-TA-N-850	0.1 M KOH	0.98	-	−6	1
Fe-N/C	0.1 M KOH	0.923	-	−6	2
Fe-N-CNF	0.1 M KOH	0.93	0.81	−5.12	3
$Fe_{1-x}S$/N,S MGCS	0.1 M KOH	0.97	0.91	−6	4
FeS/G	0.1 M KOH	1.0	0.845	−5	5
$Co_{0.5}Fe_{0.5}S$@N-MC	0.1 M KOH	0.913	0.808	−6	6
Co_9S_8/N,S-CNS	0.1 M KOH	0.90	-	−5	7
CoS NWs@NSC-2	0.1 M KOH	0.93	0.84	-	8

Supplementary References

1. Wei, J.; Liang, Y.; Hu, Y.; Kong, B.; Simon, G.P.; Zhang, J.; Wang, H. A versatile iron–tannin-framework ink coating strategy to fabricate biomass-derived iron carbide/Fe-N-carbon catalysts for efficient oxygen reduction. Angew. Chem. Int. Ed. **2016**, *55*, 1355–1359.
2. Lin, L.; Zhu, Q.; Xu, A.W. Noble-metal-free Fe–N/C catalyst for highly efficient oxygen reduction reaction under both alkaline and acidic conditions. J. Am. Chem. Soc. **2014**, *136*, 11027–11033.
3. Wu, Z.Y.; Xu, X.X.; Hu, B.C.; Liang, H.W.; Lin, Y.; Chen, L.F.; Yu, S.H.., Iron carbide nanoparticles encapsulated in mesoporous Fe-N-doped carbon nanofibers for efficient electrocatalysis. Angew. Chem. **2015**, *127*, 8297–8301.
4. Xiao, J.; Xia, Y.; Hu, C.; Xi, J.; Wang, S. Raisin bread-like iron sulfides/nitrogen and sulfur dual-doped mesoporous graphitic carbon spheres: a promising electrocatalyst for the oxygen reduction reaction in alkaline and acidic media. J. Mater. Chem. A **2017**, *5*, 11114–11123.
5. Gautam, J.; Tran, D.T.; Singh, T.I.; Kim, N.H.; Lee, J.H. Mesoporous iron sulfide nanoparticles anchored graphene sheet as an efficient and durable catalyst for oxygen reduction reaction. J. Power Sources **2019**, *427*, 91–100.
6. Shen, M.; Ruan, C.; Chen, Y.; Jiang, C.; Ai, K.; Lu, L. Covalent entrapment of cobalt–iron sulfides in N-doped mesoporous carbon: Extraordinary bifunctional electrocatalysts for oxygen reduction and evolution reactions. ACS Appl. Mater. Interfaces **2015**, *7*, 1207–1218.
7. Wu, C.; Zhang, Y.; Dong, D.; Xie, H.; Li, J. Co 9 S 8 nanoparticles anchored on nitrogen and sulfur dual-doped carbon nanosheets as highly efficient bifunctional electrocatalyst for oxygen evolution and reduction reactions. Nanoscale **2017**, *9*, 12432–12440.
8. Han, C.; Li, Q.; Wang, D.; Lu, Q.; Xing, Z.; Yang, X. Cobalt sulfide nanowires core encapsulated by a N, S codoped graphitic carbon shell for efficient oxygen reduction reaction. Small **2018**, *14*, 1703642.
